# Sulfated glycosaminoglycans in human vocal fold lamina propria^☆^

**DOI:** 10.1016/j.bjorl.2016.05.003

**Published:** 2016-06-02

**Authors:** Sung Woo Park, Gustavo Polacow Korn, Elsa Yoko Kobayashi, João Roberto Maciel Martins, Noemi Grigoletto De Biase

**Affiliations:** a Universidade Federal de São Paulo (UNIFESP/EPM), Escola Paulista de Medicina, Departamento de Otorrinolaringologia e Cirurgia de Cabeça e Pescoço, Setor de Laringe e Voz, São Paulo, SP, Brazil; b Universidade Federal de São Paulo (UNIFESP/EPM), Escola Paulista de Medicina, Departamento de Bioquímica, Divisão de Biologia Molecular, São Paulo, SP, Brazil; c Universidade Federal de São Paulo (UNIFESP/EPM), Escola Paulista de Medicina, Departamento de Medicina, Divisão de Endocrinologia e Metabologia, São Paulo, SP, Brazil; d Pontifícia Universidade Católica de São Paulo (PUC-SP), São Paulo, SP, Brazil

**Keywords:** Larynx, Glycosaminoglycans, Vocal folds, Laringe, Glicosaminoglicanas, Pregas vocais

## Abstract

**Introduction:**

The distribution, concentration and function of glycosaminoglycans in the various vocal fold tissues are still unclear.

**Objective:**

To evaluate the distribution and concentration of sulfated glycosaminoglycans in different layers of the human vocal fold according to gender and age.

**Methods:**

We used 11 vocal folds obtained from cadavers (7 men and 4 women) with no laryngeal lesion, less than 12 h after death, and aged between 35 and 98 years. The folds underwent glycosaminoglycans extraction from the cover and ligament, and post-electrophoresis analysis. Data were compared according to the layer, age and gender.

**Results:**

The concentration of dermatan sulfate was significantly higher in all layers. No differences were observed in the total concentrations of glycosaminoglycans in layers studied according to gender. It is significantly lower in the cover of individuals aged below 60 years.

**Conclusion:**

Dermatan sulfate, chondroitin sulfate, and heparan sulfate were observed in the human vocal folds cover and ligament of both genders, with the concentration of dermatan sulfate being significantly higher in all layers. Glycosaminoglycans concentration on the cover is significantly lower in individuals below 60 years compared with elderly.

## Resumo

**Introdução:**

A distribuição, concentração e função dos glicosaminoglicanos nos diversos tecidos da prega vocal ainda não está esclarecida.

**Objetivo:**

Avaliar a distribuição e concentração dos glicosaminoglicanos sulfatados nas diferentes camadas da prega vocal humana de acordo com o sexo e a idade.

**Método:**

Foram utilizadas 11 pregas vocais obtidas de cadáveres (sete homens e quatro mulheres) sem lesão de laringe, com menos de 12 horas de óbito e com idade entre 35 e 98 anos. As pregas foram submetidas à extração de glicosaminoglicanos da cobertura e ligamento e leitura pós eletroforese. Os dados foram comparados segundo a camada, idade e sexo.

**Resultados:**

A concentração de Dermatan sulfato foi significativamente maior em todas as camadas. Não foram observadas diferenças nas concentrações totais de glicosaminoglicanos nas camadas estudadas quanto ao gênero. É significantemente menor em indivíduos com idade abaixo de 60 anos na cobertura.

**Conclusão:**

Dermatam sulfato, condroitim sulfato e heparam sulfato foram observados na cobertura e ligamento de pregas vocais humanas, de ambos os sexos, sendo a concentração de dermatam sulfato significativamente maior em todas as camadas. A concentração de glicosaminoglicanos na cobertura é significativamente menor em indivíduos abaixo de 60 anos em comparação com idosos.

## Introduction

The vocal folds are adapted for speech function. The vibration of the vocal folds depends on its histological structure, especially of the lamina propria (LP), stratified in the free edge region in three layers: superficial, intermediate and deep. All of them consist of cells and extracellular matrix, differing qualitatively and quantitatively from each other, especially as to the fibrous proteins (collagen and elastic fibers) and interstitial proteins.^1^

Among the interstitial proteins, the glycosaminoglycans (GAGs), proteoglycans (PG), and non-collagenous glycoproteins are notable. They influence viscosity, hydration and tissue volume.^2^, ^3^ The differences in the arrangements and quantities of components of the extracellular matrix, and also the different interactions of these components with each other and the cells, are dynamically adjusted to the functional demands of each tissue.^4^, ^5^, ^6^ In the case of the vocal folds, this functional demand is the production of the mucosal wave and, consequently, the vibration of the tissue and the formation of the sound wave.^7^

Pawlak et al.^2^ were the first to study the PGs and GAGs in vocal fold LP. The expression of hyaluronic acid receptor, keratan sulfate, chondroitin sulfate, heparan sulfate PG, and decorin was observed in various regions, as well as the cell types in the LP.

Paulsen et al.^8^ observed a reduction of sulfated GAGs in the tendons (extremities) of the vocal ligament. A greater loss of GAG in the vocal ligament tendon was observed with aging. These structures thus lose their ability to retain water and the viscoelasticity of the vocal fold is impaired. Hammond et al.,^9^ used an indirect method to compare tissue that is incubated or not incubated with hyaluronidase and observed that the concentration of hyaluronic acid (HA) in the LP was higher in males compared to females. The distribution also varied according to the layers and gender, being more evenly distributed among males and more concentrated in the deeper layers in females.^10^ Using a direct method, Lebl et al.^11^ and Korn et al.^12^ found a higher concentration of HA in female LP in relation to male and a trend for a decrease with aging. Since bovine testicular hyaluronidase is not specific for HA, it may have broken other GAGs present in the LP, such as chondroitin sulfate, which would explain the difference found in the two studies if there were also differences in the concentrations of the other interstitial proteins.

Knowledge of the distribution and concentration of interstitial macromolecules that have different characteristics, features and effects on the functionally stratified LP layers, will help to understand the normal physiology of phonation, aging and in pathological processes that have an impaired mucosal wave.

Thus, the objective of this study is to investigate the distribution and concentration of sulfated GAGs in the different layers of the vocal fold according to gender and age.

## Methods

### Prospective experimental study

We used 11 vocal folds obtained in necropsies of 11 cadavers (7 men and 4 women) with no laryngeal lesion involved in the cause of death, procured within 12 h of death, and aged between 35 and 98 years. The project was approved by the Research Ethics Committee of a university (CEP 1203/07). Larynges were removed from the cadavers, and taken under refrigeration to the laboratory where they were dissected. The pieces were evaluated with the use of a camera and video system with a 25× magnification, and those in which any injuries or structural changes, such as polyps, furrows, leukoplakia were observed were excluded from this study.

A vocal fold from each individual was used at random. The vocal folds underwent decortication with the help of the video system. Three fragments were isolated: cover (epithelium and superficial layer of the LP), ligament (intermediate and deep layers of the LP) and muscle. From these, the cover and ligament were used.

For the GAG extraction from tissues, the fragment of the given layer of the vocal fold was perforated with a scalpel, homogenized, and dehydrated in acetone for 18 h at room temperature. Then, excess acetone was removed by centrifugation, 4000 × *g*, 15 min, and the precipitate dried in an oven. For each 1 mg of dry powder, 100 μL of maxatase, 4 mg/mL in 0.05 M Tris–HCl + 1 M NaCl were added, pH 8, and incubated at 60 °C for 18 h. After incubation, protease was inactivated by heating at 100 °C, 20 min. To the solution, 90% trichloroacetic acid was added under cooling to a final concentration of 10%. The solution was homogenized and left at 4 °C for 10 min. After this period, it was centrifugated at 4000 × *g* for 15 min. The supernatant was separated and added to absolute ethanol (3 volumes), and the final solution maintained at −20 °C for at least 3 h. After this, it was centrifugated at 10,000 × *g*, 30 min, and the obtained precipitate was dried under vacuum and stored at −20° C until analysis.^13^, ^14^

**Figure 1:**
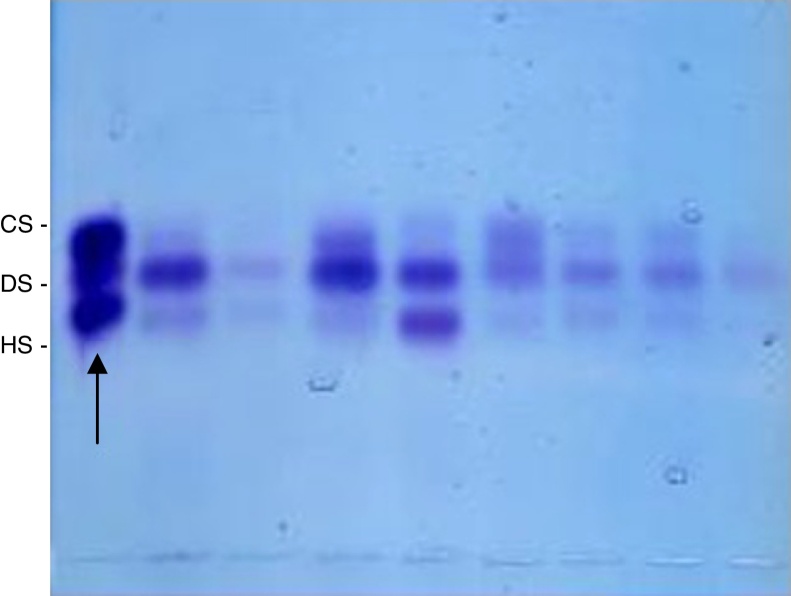
Bands obtained following 0.1% toluidine blue staining in a solution of 1% acetic acid, and 50% ethanol and destaining with 1% acetic acid and 50% ethanol, which were quantified by densitometry. The standard is on the left of the image (arrow). CS, condroitin sulfate; DS, dermatan sulfate; HS, heparan sulfate.

Then, the precipitate obtained in the previous procedures was resuspended again in distilled water (10 μL per 1 mg of dry tissue), and 5 μL of this solution were applied in agarose gel blades, 1,3-diaminopropanoacetate buffer, pH 9, and underwent electrophoresis (5 V/cm, 1 h, under refrigeration) for separation of various GAGs.^10^, ^11^ As standard, a mixture of GAGs 1 μg/mL (heparan sulfate + dermatan sulfate + chondroitin sulfate) was used. Following electrophoresis, the gel was dried under ventilation at room temperature, and stained with 0.1% toluidine blue in 1% acetic acid and 50% ethanol. After 10 min, the gel was destained with acetic acid solution 1% and 50% ethanol; dried at room temperature, and the obtained bands, quantified by densitometry (Fig. 1). The concentration of GAG was expressed in μg/g of dry tissue.^15^

Data was statistically analyzed. To compare the values of GAG concentration according to gender and age the nonparametric Mann–Whitney test was used. To compare the values of GAG concentration in the different layers of the vocal fold the Wilcoxon nonparametric test was used. Descriptive statistics of quantitative variables were performed: concentration by layer through the mean and median; and standard deviation, minimum and maximum to show variability. Also, the Analysis of Variance with Repeated Measures (univariate) was used as the observations in the different layers are related to the same individual. To compare the concentration of GAG among the layers of the vocal fold, the covariance matrix of the observations of the same individual should have a specific form. When this particular form was not found, and because of the small sample size, the test was corrected based on Huynh–Feldt correction. Comparisons among concentrations were made using the Bonferroni test. The existence of interaction (interaction effect) in the results was also checked, i.e., different behaviors according to another variable. For all tests, a significance level of 5% (*p* ≤ 0.05) was considered.

## Results

The present study evaluated Vocal Folds (vocal folds) of 11 subjects, 4 female and 7 male. Five subjects were 60 years of age or younger and the other 6 individuals were over 60 years of age.

There was no significant difference between the cover and the ligament with respect to the concentration of sulfated GAGs (Table 1).

**Table 1:** GAG concentration in the vocal fold cover and ligament.

Variables	GAGs (μg/g dry weight)
*Cover*	
Mean ± standard deviation	302.7 ± 224.9
Median (Minimum − Maximum)	217.3 (83.9–802.9)
Total	11

*Ligament*	
Mean ± standard deviation	379.3 ± 198.6
Median (minimum − maximum)	322.5 (117–681)
Total	11

*p* value^a^	0.334

a *p* value of nonparametric paired-sample Wilcoxon signed-rank test.

There was no significant difference in the concentration of sulfated GAGs in the layers of cover and ligament according to gender (Table 2).

**Table 2:** GAG concentration according to gender in the cover and ligament.

Variables per gender	Female GAGs (μg/g dry weight)	Male GAGs (μg/g dry weight)	*p*-value^a^
*Cover*			
Mean ± standard deviation	346.8 ± 307	277.5 ± 187.4	0.571
Median (minimum − maximum)	224.4 (135.7–802.9)	182.2 (83.9–535.2)	
Total	4	7	

*Ligament*			
Mean ± Standard deviation	490.1 ± 222.3	316 ± 167.9	0.257
Median (minimum − maximum)	554.9 (169.7–681)	280.6 (117–632.1)	
Total	4	7	

a *p*-value of nonparametric Mann–Whitney test.

**Figure 2:**
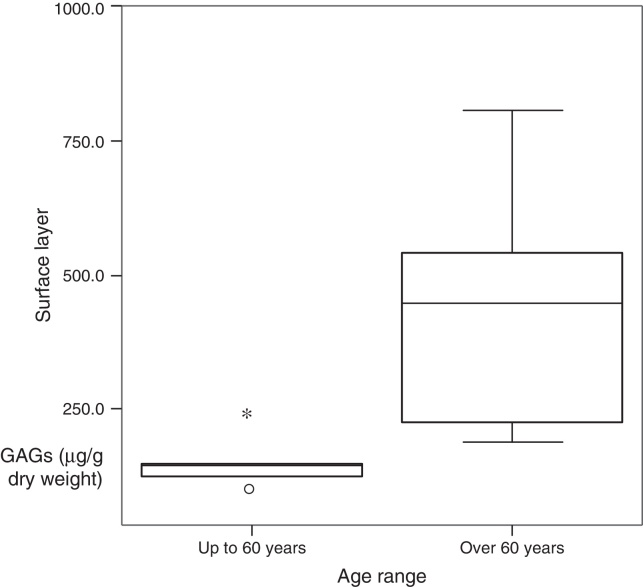
Box-plot of cover according to age range.

Comparing the concentration of sulfated GAGs as to age range, in the different layers, there was significant difference in the cover. Patients over 60 years have significantly higher values than those 60 years and younger (Table 3 and Fig. 2).

**Table 3:** GAG concentration according to the age range in: cover and ligament.

Variables per age range	Over 60 years	Up to 60 years	*p*-value
*Cover*			
Mean ± standard deviation	436.6 ± 226.6	141.9 ± 54.7	0.018^a^
Median (minimum − maximum)	441.1 (182.2–802.9)	135.7 (83.9–231.4)	
Total	6	5	

*Ligament*			
Mean ± standard deviation	410.3 ± 201.2	342.1 ± 211.9	0.584
Median (minimum − maximum)	429.2 (117–632.1)	236.1 (169.7–681)	
Total	6	5	

a *p*-value of the nonparametric Mann–Whitney test.

When comparing the concentration of CS, DS and HS in the cover and ligament, there was a higher concentration of DS in the different layers, but with no significant difference in the comparison between the layers (Table 4, Table 5).

**Table 4:** GAG concentration (CS, DS e HS) in the different layers of the vocal fold.

Variables per concentration	CS	DS	HS	*p*-value^a^
*Cover*				
Mean ± standard deviation	29.9 ± 28.8	233.7 ± 187.8	39.1 ± 36.7	<0.001
Median (minimum − maximum)	19.1 (0–82.5)	178.9 (70.2–670.1)	27.2 (0–110.5)	
Total	11	11	11	

*Multiple comparisons*				
CS × DS	0.009			
CS × HS	1.000			
DS × HS	0.009			

*Ligament*				
Mean ± standard deviation	45.6 ± 54.4	273.8 ± 119	60 ± 55.3	<0.001
Median (minimum − maximum)	23.9 (2.9–156)	289.2 (103.5–444.3)	48.5 (5.7–161.1)	
Total	11	11	11	

*Multiple comparisons*				
CS × DS	0.009			
CS × HS	1.000			
DS × HS	0.009			
*p*-value^b^	0.477	0.374	0.657	

a *p*-value of nonparametric sample-paired Wilcoxon signed-rank test.

b Multiple comparisons: *p* value of nonparametric sample-paired Wilcoxon signed-rank test.

**Table 5:** GAG concentration according to gender and age range in: cover and ligament.

	Females		Males	
	Up to 60 years	Over 60 years	Up to 60 years	Over 60 years
*Cover*				
Mean ± standard deviation	183.6 ± 67.7	510.1 ± 414.1	114.2 ± 28.5	399.9 ± 151.9
Median (minimum − maximum)	183.6 (135.7–231.4)	510.1 (217.3–802.9)	118.2 (83.9–140.5)	441.1 (182.2–535.2)
Total	2	2	3	4

*Ligament*				
Mean ± standard deviation	425.4 ± 361.5	554.9 ± 26.7	286.6 ± 113.4	338 ± 215.2
Median (minimum − maximum)	425.4 (169.7–681)	554.9 (536–573.8)	236.1 (207.1–416.4)	301.5 (117–632.1)
Total	2	2	3	4

Table 5 refers to the GAG concentration according to the age and gender in the layers of cover and ligament. Statistical analysis was not possible due to the number of vocal folds in each category.

## Discussion

Some components of the extracellular matrix in the vocal folds are better known and studied, such as elastin and collagen, and more recently the HA. However, other components of the extracellular matrix, such as GAGs and PGs, have not been studied for their role and importance in the physiology of the human vocal folds.

Thus, considering the physiology of vocal fold in the production of voice and the distinctive properties already known, this study examined the presence and concentration of sulfated GAGs in human vocal fold: on the cover, in which the superficial layer of LP predominates, and on the ligament. The cover and the ligament have distinct functions in the mucosal wave formation, and a typical histological constitution compared to fibrous proteins, elastin and collagen.^1^, ^7^ The separation between coverage and ligament, and between ligament and muscle, is easily performed by dissection with magnification.

The total concentration of sulfated GAGs in the different layers was compared, and showed similar distribution (Table 1). There was also no significant difference, in the different layers and the genders (Table 2). Other authors using a different technique, without separating the layers, also found no difference in the total GAG in relation to gender.^16^, ^17^

However, considering the age, a statistically significant difference between the concentrations of GAGs in the cover was seen, with higher values in individuals over 60 years (Table 3). The highest concentration of GAG in the elderly may also be related to the decrease of other substances in the vocal folds, such as the HA itself, as observed by Lebl et al.^11^ Korn et al.,^12^ because its concentration is calculated in terms of μg/dry weight of the vocal fold material. The ligament shows no statistically significant difference in the concentration of GAG, by either gender or age (Table 2, Table 3). Paulsen et al.^8^ observed a reduction of sulfated GAGs in the vocal ligament tendons, that is, on the ends of the ligament, as well as more pronounced loss of GAG in tendon vocal ligament with the aging process.

Hammond et al.^9^ in a study that looked at the concentration of elastin found in vocal folds with age and gender, found no significant difference in terms of gender, but an important and significant difference as for the age of patients. Adult vocal folds showed less elastin than the elderly. However, this increase in elastin density does not necessarily mean an increase in vocal folds elasticity, since the fibers observed in the elderly had a changed structure.

Thus, change of the elastin structure, the concentration of HA and sulfated GAGs may be related to tissue aging with consequent repercussions on the production of mucosal wave and voice.

When the cover and the ligament of the three types of GAGs (chondroitin, heparan and dermatan sulfate) were compared separately, there was no significant difference. However, both on the cover and the ligament, the comparison between the types of GAGs shows significantly higher concentration of dermatan sulfate (Table 4, Table 5). Pawlak et al.^2^ carried out a study on the presence and distribution of GAGs and PG in the vocal fold with anti-keratan sulfate, anti-CS PG, anti-HS PG and antidecorin. Data were quantified in accordance with staining compared to normal skin sample, at 0–3+. The authors observed keratan sulfate and decorin (which can bind covalently to QS or DS) in fibrillar components in the LP. Decorin predominated in the surface layer (2+) in relation to the deep layers and QS in the deep ones relative to the surface. CS was observed only in the LP cell cytoplasm (2+). HS PG were observed in the basal lamina and blood vessels, as well as in the cytoplasm of macrophages, and probably fibroblasts (2+). Given the results, the authors point to the probable importance of fibromodulin (covalently bound to QS in the deep layers and decorin in the surface, both related to the regulation of the type and size of the produced collagen. The authors did not study DS, the GAG found in greater concentration in this study. Its presence in large amounts in the two layers of the vocal fold should be better understood as well as the higher amount of GAGs observed with aging.

Data from this study point to the need for further research with larger numbers of individuals and not just sulfated GAGs, but also the different types of PGs formed from its connection to the different protein structures, to understand the function of each GAG and PG in the physiology of the vocal fold as a sound source.

## Conclusions

1. Dermatan sulfate, chondroitin sulfate and heparan sulfate were observed in the cover and ligament of human vocal folds, of both genders, and the concentration of dermatan sulfate is significantly higher in all layers.
2. There were no significant differences in the concentrations of GAGs between coverage and ligament according to gender.
3. The concentration of GAG in the cover is significantly lower in individuals below 60 years compared to the elderly.

## Conflicts of interest

The authors declare no conflicts of interest.
